# Nonlinear increase, invisibility, and sign inversion of a localized fs-laser-induced refractive index change in crystals and glasses

**DOI:** 10.1038/s41377-020-0298-8

**Published:** 2020-04-20

**Authors:** Jerome Lapointe, Jean-Philippe Bérubé, Yannick Ledemi, Albert Dupont, Vincent Fortin, Younes Messaddeq, Réal Vallée

**Affiliations:** Centre d’Optique, Photonique et Laser, 2375 Rue de la Terrasse, Université Laval, G1V 0A6 Québec, QC Canada

**Keywords:** Laser material processing, Integrated optics, Nonlinear optics, Photonic devices

## Abstract

Multiphoton absorption via ultrafast laser focusing is the only technology that allows a three-dimensional structural modification of transparent materials. However, the magnitude of the refractive index change is rather limited, preventing the technology from being a tool of choice for the manufacture of compact photonic integrated circuits. We propose to address this issue by employing a femtosecond-laser-induced electronic band-gap shift (FLIBGS), which has an exponential impact on the refractive index change for propagating wavelengths approaching the material electronic resonance, as predicted by the Kramers–Kronig relations. Supported by theoretical calculations, based on a modified Sellmeier equation, the Tauc law, and waveguide bend loss calculations, we experimentally show that several applications could take advantage of this phenomenon. First, we demonstrate waveguide bends down to a submillimeter radius, which is of great interest for higher-density integration of fs-laser-written quantum and photonic circuits. We also demonstrate that the refractive index contrast can be switched from negative to positive, allowing direct waveguide inscription in crystals. Finally, the effect of the FLIBGS can compensate for the fs-laser-induced negative refractive index change, resulting in a zero refractive index change at specific wavelengths, paving the way for new invisibility applications.

## Invisible and miniaturized laser-inscribed photonic circuits

Energy gap changes in photonic materials induced by femtosecond laser pulses could lead to new invisibility and miniaturized applications. Jerome Lapointe and colleagues at Canada’s Laval University conducted theoretical and experimental investigations to study the effects of inducing ‘band gap shifts’ within crystals and glasses when a femtosecond laser is used to inscribe optic waveguides inside them. The new technology led to nanoscale changes that supported the inscription of smooth, bendy channels that can be used to efficiently direct light through them while minimizing energy loss. This is a significant step to meet the growing demand for miniaturized photonic and quantum circuits. These channels also become invisible to the naked eye when red light is shone through them, paving the way to transparent photonics applications, which can be placed on phone screens and car windshields.

## Introduction

Femtosecond (fs) laser inscription in transparent materials has unique advantages^1,2^. One of the most relevant advantage is the micrometer-scale processing of complex three-dimensional structures, owing to the nonlinear nature of the laser absorption that precisely confines structural changes to the focal volume. However, a severe limitation of fs-laser inscription is related to the relatively low photoinduced refractive index contrast that is achievable^3,4^. In particular, the miniaturization of many fs-laser-processed photonic devices is limited by the minimum bend radius of waveguides, which in turn depends on the magnitude of the induced refractive index contrast. Another important limitation is the decrease in the refractive index that occurs in most crystals^5^ and in a wide variety of glasses^6–8^. In fact, many applications, such as waveguide lasers^9^, electro-optic modulators^10^, and frequency converters^11^, require multi-scan-depressed cladding structures, which complicate or impede the fabrication or their guiding circuits.

In most applications of photonics, the propagating wavelengths are far from the material resonances to minimize the optical losses; the fiber-optic communication window around 1550 nm in fused silica is a good example. Away from resonances, the compaction and rarefaction of the structural network (affecting the number of charged particles per volume unit) and other mechanisms such as color centers, a change in the fictive temperature, and defect-induced density changes largely dominate the refractive index change in fs-laser-processed photonic circuits^1,6,12^. However, for propagating wavelengths approaching the electronic resonance, we show that the refractive index change exponentially increases owing to a fs-laser-induced band-gap shift (FLIBGS). For the first time, to the best of our knowledge, the effect of the FLIBGS in transparent materials is studied. Note that the propagating wavelengths are studied near the resonance, which are not to be confused with the wavelength used to process the material with the fs laser (producing the FLIGBS) that is far from the resonance (fixed at 795 nm in this work). For the remainder of the paper, the stated wavelengths refer to light propagating in the waveguides.

Using this FLIGBS phenomenon, we demonstrate that the sign of the refractive index contrast can be inverted, which allows for the direct inscription of smooth waveguides (i.e., type I-positive refractive index change) in crystals. This type of inscription has several advantages over structures based solely on the stress induced by damage tracks traditionally inscribed in crystals^5^ or glasses^6–8^ using high-energy laser pulses (i.e., the so-called type III modifications^13^). Moreover, preliminary results show potential invisibility applications. The opposition between the fs-laser-induced negative refractive index change and the positive refractive index change due to the FLIBGS can result in a zero refractive index change at specific wavelengths, which theoretically enable invisibility. While invisibility cloaking has gained much attention in recent years^14,15^, mostly due to metamaterials^16,17^, the FLIGBS mechanism demonstrates a new concept for the direct fabrication of invisible structures, paving the way for new invisibility applications. Finally, we demonstrate a lower propagation loss in tightly curved waveguides mostly due to the high refractive index change induced by the FLIBGS, which creates opportunities for miniaturized devices. Supported by theoretical analysis, we experimentally demonstrate waveguide bends with a submillimeter radius of curvature, which is an important improvement over the minimum 10-mm radius reported previously^3,18^. It is somehow implicit that the use of the FLIBGS results in a practical range of applications characterized by a narrow spectral band near the resonance of the material. Surprisingly, the FLIBGS affects the refractive index over a certain region beyond the absorption edge bandwidth in the highly transparent region, which extends the application range. Moreover, since electronic bandgaps lying in the ultraviolet, visible, and infrared regions can be found in different materials^19^, the FLIBGS has great potential for the entire spectral band in photonics.

## Results

### FLIBGS theory and experiment

The ultrafast laser-induced refractive index change of transparent materials is a complex phenomenon that relies on different physical processes. First, a physical rearrangement of the structural network was observed^1,20^. It is believed that the densification induced by various complex phenomena, such as a fast temperature change^21,22^ and plasma shock waves^23,24^, has a great impact on the fs-laser-induced refractive index change. Another process is related to the variation in the absorption spectrum through the Kramers–Kronig relations^25^. Increased absorption due to photoinduced defects such as color centers^26,27^ produced by self-trapped excitons^28^ leads to a variation in the refractive index. Since most of the defects can be annealed while a partial refractive index change remains^29^, defects can only partially explain the laser-induced refractive index change.

**Fig. 1:**
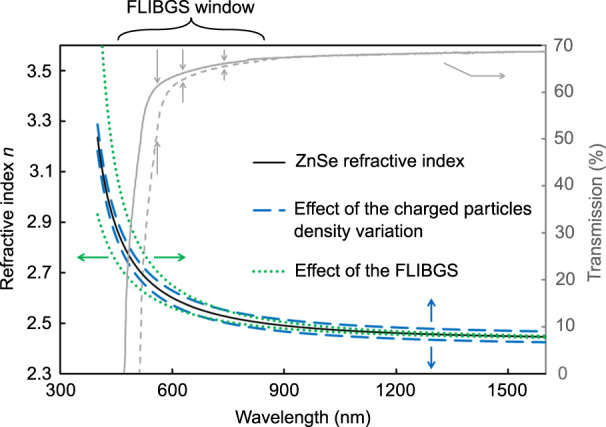
Left axis: refractive index of ZnSe as a function of the propagating wavelength using the Sellmeier coefficient from ref. ^33^ (black curve). Potential effects of the fs-laser-induced variation in the number of charged particles per volume unit (blue dashed curve) and the FLIBGS (green dotted curve), based on Eq. 4. Right axis: ZnSe transmission spectrum (gray curve). The double gray arrows show the potential absorption variation in the FLIBGS window if a shift in the absorption edge occurs (dashed gray curve)

To date, no one has explicitly studied the effect of the FLIBGS on the refractive index. A first way of approaching this problem is via the Kramers–Kronig relations, relating the refractive index to the absorption coefficient *α* integrated over frequency:^30^

1 $$n\left( \omega \right) = 1 + \frac{\pi }{c}{{\wp}} \int\nolimits_{0}^{+ \infty} {\frac{{\alpha \left( {\omega^{\prime}} \right)}}{{\omega^{\prime2} - \omega^{2}}}} d\omega ^{\prime}$$

where *c* is the speed of light, *ω*′ is the angular frequency variable running through the whole integration range, and ℘ denotes the Cauchy principal value. Clearly, from this relation, a change in the absorption *α*(*ω*′) curve will in turn affect *n*(*ω*). To illustrate this effect, Fig . 1 shows the transmission spectrum through a zinc selenide (ZnSe) crystal with a thickness of *d* = 1 mm (gray curve, associated with the right axis). When a band-gap shift occurs, the absorption edge (near the electronic resonance) of the transmission spectrum shifts horizontally. For illustrative purposes, a dashed gray curve has been added to represent the shifted spectrum. At a wavelength near the absorption edge (where the transmission slope is significant, denoted by the FLIBGS window in Fig. 1), the shift greatly affects the absorption (double gray arrows) and thus the refractive index.

Equation 1 is not convenient to use experimentally since it requires measurements over a very wide spectral band. Alternatively, the Lorentz dispersion relation with the Clausius–Mossotti form allows one to express the refractive index in terms of the number of charged particles per volume unit *N*_k_^31^:

2 $$\frac{{3\left( {\bar n^2 - 1} \right)}}{{\left( {\bar n^2 + 2} \right)}} = \mathop {\sum}\limits_{\mathrm{k}} {\frac{{4\pi N_{\mathrm{k}}\varepsilon _{\mathrm{k}}^2/m_{\mathrm{k}}}}{{\omega _{0{\mathrm{k}}}^2 - \omega ^2 + i\gamma \omega }}}$$

where $\bar n$ is the complex refractive index and *m*_k_ is the mass of particle *k* with charge *ε*_k_. The number of charged particles per volume unit *N*_k_, the resonance frequency $\omega _{0k}$, and the damping coefficient $\gamma$ are the only terms that can potentially be modified using fs-laser irradiation. The real part of the refractive index *n* can be experimentally obtained using the well-known Sellmeier equation, an empirical equation related to Eq. 2, as a function of the wavelength λ:

3 $$n^2 = A + \mathop {\sum}\limits_{\mathrm{k}} {\frac{{B_{\mathrm{k}}\lambda ^2}}{{\lambda ^2 - C_{\mathrm{k}}^2}}}$$

where the first (*A*) and second (*k* = 1) terms of this series represent the contributions to the refractive index due to the higher- and lower-energy bandgaps of electronic absorption, respectively, whereas the remaining terms (*k* > 1) account for a refractive index modification due to lattice resonance^32^. Equation 2 suggests that *B*_k_ is closely linked to the number of charged particles per volume unit *N*_k_ and *C*_k_ to the resonance frequency $\omega _{0k}$ (or wavelength *λ*_0k_). Note that the damping is not considered in the Sellmeier equation (also neglected in this work) since it is only significant in the close vicinity of the resonances. In addition, note that the damping is related to the absorption coefficient *α* and the Cauchy principal value ℘ in Eq. 1. Since the bandgap, absorption edge, and resonance frequency of a material are directly connected, the three models (using Eq. 1, Eq. 2, or Eq. 3) are similar in terms of studying the FLIBGS.

To experimentally study the FLIBGS, the following modified Sellmeier empirical equation that includes the effect of fs-laser irradiation is suggested:

4 $$n_{irr}^2 = A + \mathop {\sum}\limits_k {\frac{{\left( {B_{\mathrm{k}} + dN_{\mathrm{k}}} \right)\lambda ^2}}{{\lambda ^2 - \left( {C_{\mathrm{k}} + d\lambda _{\mathrm{k}}} \right)^2}}} \approx A + \frac{{\left( {B_1 + dN_1} \right)\lambda ^2}}{{\lambda ^2 - \left( {C_1 + d\lambda _1} \right)^2}}$$

where *dN*_*k*_ is proportional to the laser-induced variation in the number of charged particles per volume unit and *dλ*_k_ is the laser-induced resonance shift (linked to the FLIGBS). The remaining terms (*k* > 1) are assumed to be negligible for wavelengths relatively close to the *λ*_1_ (or *C*_1_) electronic resonance, which is the case in this work. The fs-laser-induced refractive index contrast is Δ*n* = *n*_irr_ − *n*, where *n*_irr_ is the refractive index of the irradiated region.

For illustrative purposes, Fig. 1 shows the ZnSe refractive index curve with the Sellmeier coefficients *A* = 4, *B*_1_ = 1.90, and *C*_1_ = 336.15 nm from ref. ^33^ (black curve, associated with the left axis). The effects of the variation in the number of charged particles per volume unit (blue dashed curves with *dN*_1_ = ±0.1) and the FLIBGS (green dotted curves with *dλ*_1_ = ±30 nm), both exaggerated to clearly observe their effect over the full spectrum, are plotted. The variation in the number of charged particles per volume unit tends to vertically displace the curve (blue arrows), which affects the refractive index similarly at all wavelengths, whereas the resonance shift tends to horizontally displace the curve (green arrows), which increasingly varies the refractive index when approaching the electronic resonance at lower wavelengths.

**Fig. 2:**
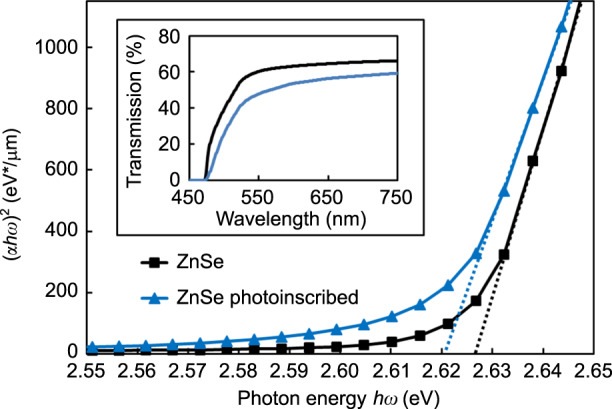
Calculating the FLIGBS value using the Tauc plot. Relationship between (*αhω*)^2^ and *hω* for a ZnSe crystal sample before (black curve) and after (blue curve) photoinscription (pulse energy of 100 nJ with a scan speed of 5mm/s). Their transmission spectra through the sample with a thickness of *d* = 1 mm (including Fresnel losses) are shown in the inset

To demonstrate the effect of the FLIBGS on the refractive index contrast of a waveguide, fs-laser inscription was performed using a Ti:sapphire laser system (Coherent RegA). The system was operated at a wavelength of 795 nm with a repetition rate of 250 kHz. The temporal FWHM of the pulses was measured to be ~65 fs at the laser output. To estimate the electronic resonance shift *dλ*_1_ induced by the fs laser in ZnSe, several lines were inscribed with a scan speed of 5 mm/s and a pulse energy of 100 nJ. The inset in Fig. 2 shows the transmission spectrum of a ZnSe sample with a thickness of *d* = 1 mm before (black curve) and after (blue curve) photoinscription, measured using an Agilent Cary 5000 UV–vis–NIR spectrophotometer. Unfortunately, uniform irradiation over a 1-mm^3^ volume would take weeks. Therefore, 3300 lines were inscribed with a lateral displacement of 3 μm to form a layer (1 cm^2^), and 7 layers were inscribed with a vertical displacement of 10 μm, from a depth of 40–100 μm. The beam was focused beneath the surface of the sample using a 100× (1.25 NA) oil immersion microscope objective. The immersion oil refractive index (1.5) was beneficial for reducing the high aberration generated by the ZnSe refractive index (approximately 2.5 at 795 nm). However, it was impossible to write deeper due to the aberration and closer to the surface due to bubble formation in the oil.

The shift in the absorption edge in BaAlBO_3_F_2_ and borosilicate glasses has been observed by two other groups, but has not been investigated^34,35^. It is very difficult to obtain a quantitative measurement of the electronic resonance shift *dλ*_1_ from the transmission spectrum (inset of Fig. 2). Nevertheless, the absorption spectrum provides an efficient means to assess the band structure and width of the energy bandgap of optical materials, from which the electronic resonance frequency can be inferred. The optical bandgap *E*_opt_ can be expressed according to the Tauc law^36^:

5 $$\left( {\alpha \left( \omega \right)h\omega } \right) = B\left( {h\omega - E_{\mathrm{opt}}} \right)^m$$

where *B* is a constant depending on the transition probability, *α* is the absorption coefficient, and is calculated using the expression *α* = −2.303log(*T*)/*d* (*d* is the thickness of the sample and *T* is the transmission), *ω* is the incident light angular frequency, *E*_opt_ is the width of the bandgap, and *m* = 1/2 is the refractive index characterizing the direct transition process.

From the experimental transmission spectrum, (*α*(*ω*)*hω*)^2^ can be plotted as a function of *hω* in eV, as shown in Fig. 2. The optical bandgap *E*_opt_ is obtained as the intersection of the extrapolated linear portion of the curve with the photon energy *hω* axis. The bandgap shifts from approximately 2.627–2.620 eV, which corresponds to an electronic resonance shift of *dλ*_1_ = 1.26 nm. As a comparison, in typical semiconductors (*E*_opt_ ∼10 eV) deformed using the piezospectroscopic effect, the strain-induced shift of an electronic resonance may be approximately 100 meV (*dλ*_1_ = 1.23 nm)^37^. Although this demonstrates an FLIBGS, the result is a lower bound since the sample is not irradiated over its whole volume.

### Sign inversion of refractive index contrast

**Fig. 3:**
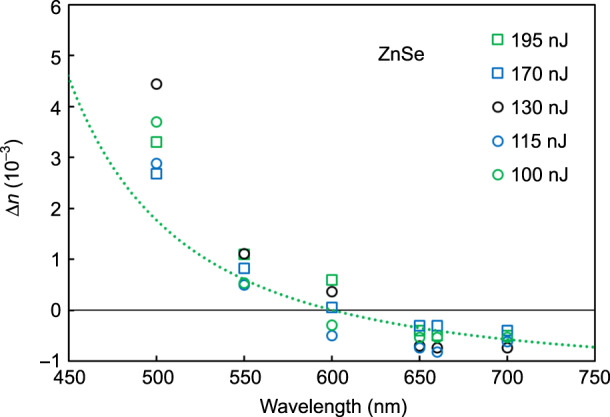
Sign inversion of the photoinduced refractive index change in ZnSe. Experimental values of the fs-laser-induced refractive index change as a function of the propagating wavelength for different laser pulse energies and the theoretical (green dotted) curve from Eq. 4 with *dλ*_1 _= 1.26 nm and *dN*_1_ = −6.5 × 10^−3^ and using the Sellmeier coefficients from ref. ^33^

Except for a few demonstrations, such as in ZnSe^38^, LiNbO_3_, and Nd:YCa_4_O(BO_3_)_3_, the refractive index change is generally negative in crystals^5^. Therefore, direct writing of waveguides in crystals is impractical. This can be explained because a positive refractive index change typically requires an increase in the material density, which is difficult to achieve in crystalline materials due to the compact structural order of the lattice, in contrast to vitreous materials with structural disorder and the existence of free space within the network. Figure 3 shows the refractive index contrast Δ*n* for waveguides inscribed in a ZnSe crystal using the same parameters mentioned previously, with pulse energies from 100 to 195 nJ, as a function of the propagating wavelength. The results demonstrate a sign inversion of the refractive index change between 550 and 650 nm, depending on the energy. To the best of our knowledge, this is the first observation of a sign inversion of refractive index contrast as a function of the propagating wavelength. Details on the refractive index contrast measurement are provided in the “Materials and methods” section.

The green dotted curve represents the refractive index change calculated using Eq. 4 with *dλ*_1_ = 1.26 nm and d*N*_1_ = −6.5 × 10^−3^ (chosen to fit the experimental value at 700 nm) and using the Sellmeier coefficients from ref. ^33^. Although the experimental points agree well with the theoretical green dotted curve, a significant discrepancy is observed at shorter wavelengths, which supports the hypothesis of an underestimation of the band-gap shift *dλ*_1_. The inaccuracy of the empirical Sellmeier coefficients from ref. ^33^ could also contribute to the error, which is supported by the large difference between the different values found in the literature^39^.

**Fig. 4:**
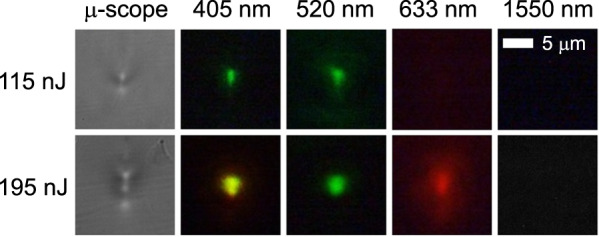
Demonstration of light guiding in a ZnSe crystal based on the FLIGBS. Microscope images of two waveguide side views (left) inscribed in ZnSe with pulse energies of 115 and 195 nJ, and their respective near-field modes imaged at different propagating wavelengths

Figure 4 shows the near-field mode profiles of the two waveguides inscribed in crystalline ZnSe with pulse energies of 115 and 195 nJ. With the 115-nJ pulses, the light is weakly confined at 520 nm and not guided at 633 nm. With the 195-nJ pulses, the light is weakly confined at 633 nm and not guided at 1550 nm. At lower wavelengths, the light is strongly confined in the waveguide at both pulse energies. The trend follows the sign inversion of the refractive index contrast. These results are of great interest, since many applications, such as waveguide lasers^9^, electro-optic modulators^10^, and frequency converters^11^, currently require multi-scan-depressed cladding structures due to the decrease in the refractive index that arises in most crystals^5^ and a wide variety of glasses^6–8^.

### Point of invisible writing

**Fig. 5:**
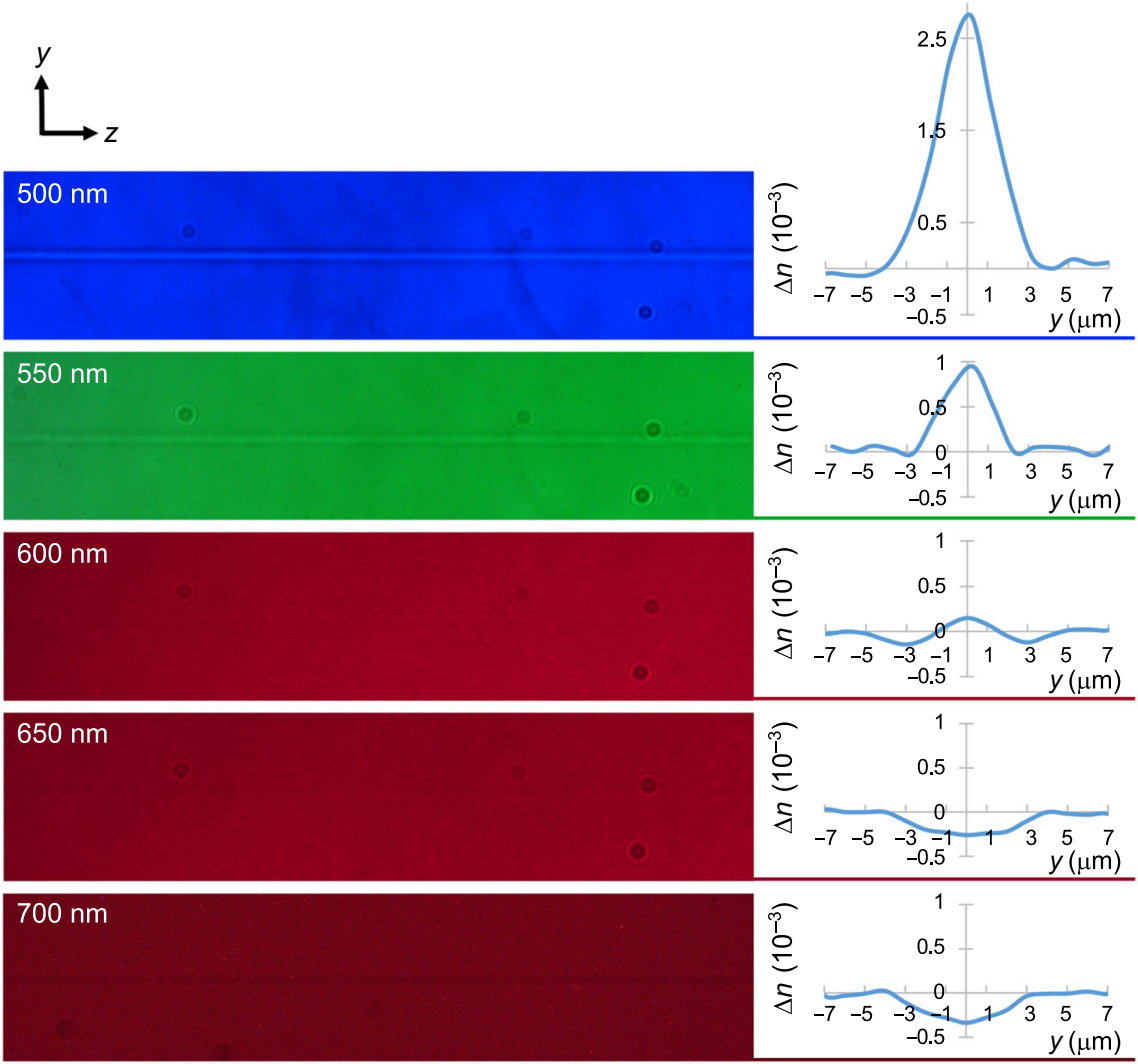
A waveguide inscribed using a fs laser in a ZnSe crystal becomes nearly invisible near 600 nm. Top views of the waveguide taken with a microscope using filters at 500, 550, 600, 650, and 700 nm (left) and their respective refractive index change profiles (right)

A peculiar phenomenon can be observed in Fig. 3 when the sign of the refractive index change is inverted as a function of the wavelength. At a specific wavelength, the refractive index contrast becomes zero, which means that the laser inscription should be invisible at this wavelength. At Δ*n* = 0, i.e., when *n* = *n*_irr_ (cf. Equations 3 and 4), the propagated light is not affected by the structural modification, which appears to be invisible. Due to the highly nonlinear effect of *dλ*_1_ compared with the effect of *dN*_1_, invisibility occurs at different wavelengths depending on the laser inscription parameters. Therefore, the FLIGBS allows for the direct inscription of invisible structures, which does not require invisibility cloaking^14–17^ to be hidden. As a preliminary experimental proof of concept, the left side of Fig. 5 shows the top view of the waveguide inscribed in ZnSe using a pulse energy of 170 nJ. The five pictures were taken with a microscope using filters at 500, 550, 600, and 650 nm. The visibility of the waveguide follows the trend of the refractive index contrast profile shown on the right of Fig. 5 (also see Fig. 3). At 500 and 550 nm, the waveguide is clearly seen. At 700 nm, the waveguide is fairly visible. At 600 and 650 nm, the waveguide is completely invisible to the naked eye and barely visible under the microscope, especially at 600 nm, where it is necessary to fine-tune the microscope focus position to make the waveguide barely visible.

However, the fs-laser-induced refractive index contrast is not perfectly uniform over the whole inscribed cross section, mostly due to the stress induced around the focal region. This prevents the refractive index contrast from being zero over the full cross-section area of the waveguide, as shown in the refractive index profile at 600 nm (see Fig. 5, right). The perfect step refractive index induced by the fs laser should theoretically enable perfect invisibility, a field that has gained much interest in the last decade^14–17^, including fs-laser-written devices in smartphone screens, such as temperature sensors^40^ and on-surface refractometric sensors for liquids^41^, that are effectively invisible to the naked eye. In these previous works^40,41^, the waveguides are undetectable to the naked eye due to the low laser-induced refractive index change, which limits the waveguide bend radii and thus the applications. Therefore, enhancing the invisibility in the visible region while increasing the refractive index change at the operating wavelength due to the FLIBGS would be of great interest. These invisible waveguide-based devices also have great potential in any see-through protection screen, such as car windshields, industrial displays, army helmets, and plane dashboards. The use of the multiscan technique or low repetition rates to avoid the heating effect^42^, and methods to minimize aberration such as using a spatial light modulator^43^ or a dual-beam technique^44^ in order to sharpen the Gaussian intensity profile should help obtain step refractive index inscriptions. Invisibility at specific wavelengths could enable interesting applications in photonic circuitry and gratings.

Note that a sign inversion of the refractive index contrast and invisibility is not possible via a type III modification (damage tracks). The negative refractive index contrast produced by voids formed due to microexplosions remains negative at any optical wavelength. Thus, invisibility can only be obtained via a negative refractive index change with a type I modification, which has been achieved in many materials^5,45^.

### High refractive index contrast allowing compact devices

An exponential increase in the refractive index contrast is observed when approaching the electronic resonance at shorter wavelengths (see Fig. 3). This feature is very interesting for the fabrication of photonic devices, such as splitters, couplers, and ring resonators, with a submillimeter size. In fact, submillimeter devices are still nearly impossible to fabricate using fs-laser writing due to the minimum waveguide bend radius limited by the refractive index contrast^3,18^. No one has used wavelengths near a material electronic resonance for photonics applications obviously because of the higher material absorption. A centimeter-long device would be too lossy to be useful. However, for very compact devices, the intrinsic material absorption becomes less problematic. In the following paragraph, we address the possible benefits of the FLIBGS for the miniaturization of fs-laser-written photonic circuits.

**Fig. 6:**
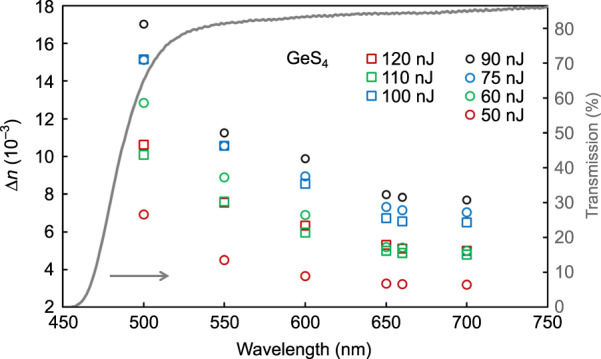
Exponential increase in the refractive index change in GeS_4_ glass as a function of the propagating wavelength for different laser pulse energies. The gray curve shows the transmission spectrum of GeS_4_ through a 1.22-mm-thick sample (including Fresnel losses). For a fixed pulse energy, a significant enhancement of the refractive index change is still observed at wavelengths within the highly transparent region

To isolate waveguide bend losses, irradiation experiments were performed on GeS_4_ glass, which has an electronic bandgap lying in the visible region, in which it is easy to photoinscribe type I waveguides^46^. Figure 6 shows the refractive index contrast Δ*n* as a function of the propagating wavelength for waveguides inscribed in GeS_4_ glass using the same parameters mentioned previously, with pulse energies from 50 to 120 nJ focused 100 μm beneath the surface using a 50× objective (Edmund Optics LWD 0.55 NA). The exponential increase in the refractive index contrast is clearly observed at short wavelengths. Positive refractive index changes up to ~1.7 × 10^−2^ are obtained at 500 nm for a pulse energy of 90 nJ. Note that this value of 1.7 × 10^−2^ is, to the best of our knowledge, the highest fs-laser-induced smooth positive type I refractive index change observed in any chalcogenide glass waveguide. The gray curve shows the transmission spectrum of the GeS_4_ glass through a 1.22-mm-thick sample (including Fresnel losses). For a fixed pulse energy, it is interesting to see that a significant enhancement of the refractive index change is still obtained at wavelengths within the highly transparent region. This extends the range of applications of FLIBGS-based devices.

**Fig. 7:**
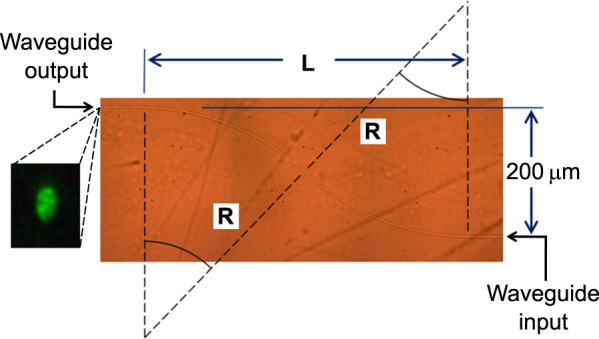
Top-view microscope image of an S-bend waveguide (with *R* = 363 μm) photoinscribed in GeS_4_ glass and (inset) its near-field mode profile at 520 nm with a width of 9.5 μm. See the dynamics of the guided light in the Supplementary Movie

To ensure a smooth inscription of the tightly curved waveguides, the scan speed was reduced to 1 mm/s. Then, 20-nJ pulses were focused 100 μm beneath the surface using a 100× oil immersion objective (1.25 NA). To isolate the curvature loss, several S-bend waveguides were written in a 6-mm-long GeS_4_ sample, as shown in Fig. 7. Six S-bend waveguides with a fixed lateral displacement of 200 μm with lengths *L* ranging from 0.5 mm (with a radius curvature *R* of 0.363 mm) to 6 mm (*R* = 45.05 mm) were written.

**Fig. 8:**
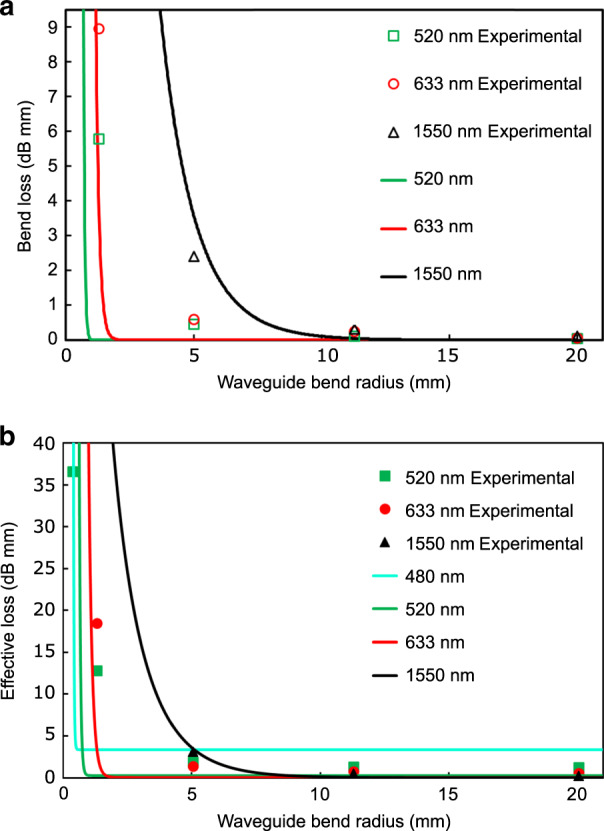
Optical losses as a function of the bend radius. **a** Waveguide bend loss and **b** effective loss (bend loss plus material absorption) at 520, 633, and 1550nm as a function of the radius curvature of an S bend photoinscribed in GeS_4_ glass. The solid curves are the theoretical curves obtained from Eq. 6. Despite the high material absorption loss, it is advantageous to use smaller wavelengths for very small bend radii (e.g., 480-nm solid curve)

The S-bend waveguides were characterized using 520-, 633-, and 1550-nm laser sources. The light injection was performed by butt-coupling with a single-mode fiber. Simply by measuring the additional loss relative to a straight waveguide written under the same conditions, the additional loss from each S bend can be isolated. The bend loss in dB/mm is obtained by dividing this additional loss over the S-bend waveguide length. The results are plotted in Fig. 8a. At 1550 nm, the results are in agreement with prior results from the literature^3,18^. For a radius curvature of 5 mm, the loss is less than 0.5 dB/mm at 520 nm, while it is over four times higher at 1550 nm. For a radius curvature of 1.3 mm, the signal is completely lost at 1550 nm, while the loss is less than 6 dB/mm at 520 nm. For a radius of curvature of 363 μm, guiding occurs only with the 520-nm light, with a bend loss of 17 ± 2 dB/mm, which seems promising for sub-millimeter-size devices, considering that 520 nm is not the optimized wavelength. Note that we have not been able to guide 520-nm light through waveguides with submillimeter bend radii in a material with a bandgap far from this wavelength, such as standard glasses (e.g., soda lime, borosilicate, and fused silica). In addition to the high refractive index contrast obtained due to the FLIBGS, the smooth type I-positive refractive index change may have an important impact on the guiding property of waveguides with submillimeter bend radii. In fact, a high refractive index contrast can be achieved with mixes of positive and negative refractive index changes or with type III (microexplosion or damage tracks) waveguides. However, the high asymmetry or roughness typically obtained from these methods induces additional losses in waveguide bends.

The experimental values can be compared with the theoretical formula of the waveguide bend loss *L*_B_ (dB/mm)^47^:

6 $$\begin{array}{l}L_{\mathrm{B}} = \frac{{2.171\pi ^{1/2}}}{{\left( {\rho R} \right)^{1/2}}}\left( {\frac{{V^4}}{{\left( {V + 1} \right)^2\left( {V - 1} \right)^{1/2}}}} \right)\\ \times \exp \left[ {\frac{{\left( {V - 1} \right)^2}}{{V + 1}} - \frac{{4R\left( {V - 1} \right)^3}}{{3\rho V^2}}\left( {\frac{{n_{irr}^2 - n^2}}{{2n_{irr}^2}}} \right)} \right]\end{array}$$

where *ρ* is the waveguide core radius and *V* is the waveguide parameter given by:

7 $$V = \frac{{2\pi \rho }}{\lambda }\left( {n_{irr}^2 - n^2} \right)^{1/2}$$

The theoretical bend loss curves for 520, 633, and 1550 nm are plotted in Fig. 8a (solid curves). The differences between the experimental values and the theoretical curves can be explained by the perturbation at the transition point (halfway point of the S bend) where the curve changes the direction of its rotation^47^, which is not taken into account in Eq. 6, and the fact that defects and waveguide roughness have more significant effects for curved segments. Moreover, Eq. 6 is an approximation for perfectly symmetrical single-mode waveguides, which is not exactly the case in our experiment. As shown in the inset of Fig. 7, the mode profile is slightly elongated, and few modes appear at smaller wavelengths. The refractive index values of the GeS_4_ glass were obtained using an interpolation from five measurements (*n* = 2.153, 2.109, 2.058, 2.044, and 2.039 at wavelengths *λ* = 532, 633, 972, 1303, and 1538 nm, respectively) using a Metricon 2010/M prism coupler.

However, the most important parameter is the total loss of such curved waveguide-based devices. The mode mismatch and Fresnel losses (at the input and output) can be easily reduced to less than 1 dB^47^ and remain the same for any S-bend size; therefore, they are not taken into account in the following loss estimation. At wavelengths far from the resonances, the propagation loss in straight waveguides can be as low as 0.01 dB/mm^1^. This waveguide propagation loss is negligible compared with the bend loss and material absorption at wavelengths near electronic resonance and even more negligible for compact devices, which is the subject of this study. Figure 8b shows the sum of the two main optical losses (bend loss and material absorption), which will be referred to as the “effective loss”, for several wavelengths as a function of the waveguide bend radius. The absorption spectrum of the GeS_4_ glass was measured using an Agilent Cary 5000 UV–vis–NIR system. Despite the higher absorption near electronic resonance, the experimental values and the theoretical curves in Fig. 8b clearly show the advantage of using wavelengths near resonance for tightly curved waveguides. For example, from the experimental measurements, a 1-mm-long optical splitter with a lateral displacement of the outputs of 400 μm, which is made of two S bends, as shown in Fig. 7, with a waveguide bend radius of 1.3 mm, exhibits an effective loss of 6.1 dB at 520 nm, while the signal is completely lost at 1550 nm. For a 1.6-mm-long splitter with a lateral displacement of the outputs of 250 μm, with a waveguide bend radius of 5 mm, the experimental effective loss is 2.16 dB at 633 nm. These relatively low losses are due to the fact that at 520 and 633 nm, the material absorption is still low, while the refractive index is significantly increased (see Fig. 6) due to the FLIBGS.

**Fig. 9:**
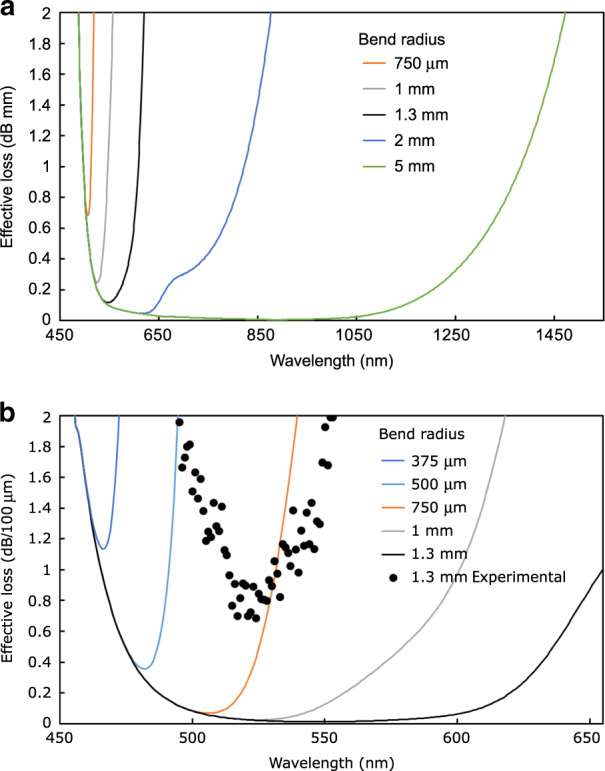
Effective loss as a function of the wavelength. **a** Theoretical effective loss (bend loss plus material absorption) as a function of the propagating wavelength for various waveguide bend radii photoinscribed in GeS_4_ glass. **b** Zoom-in of the microbend region. The experimental points (black circles) include the Fresnel, mode mismatch, and misalignment losses

Despite the differences between the experimental points and the theoretical curves in Fig. 8b, both clearly show the same trend. Therefore, the theoretical calculation can be used to provide an optimized wavelength for a specific bend radius required for a specific application. The curves in Fig. 9 show the theoretical effective loss as a function of the wavelength for different waveguide bend radii photoinscribed in GeS_4_ glass. Optimized wavelengths of 895, 620, 545, 525, 505, 480, and 467 nm are obtained for bend radii of 5, 2, 1.3, 1, 0.75, 0.5, and 0.375 mm, respectively. Moreover, as shown in Fig. 9a, low-loss compact devices made of waveguides with a bend radius of 5 mm should be achievable over a bandwidth of ∼600 nm (from ∼550 to ∼1150 nm).

Figure 9b shows the experimental effective loss measurement (black circles) using an optical spectrum analyzer (Yokogawa AQ6373B) from a white-light source (Koheras SuperK Power supercontinuum source) launched in an S-bend waveguide (as shown in Fig. 7) with a bend radius of 1.3 mm. While this method of analysis is not precise enough to obtain a reliable measurement of the losses (it also includes Fresnel, mode mismatch, and misalignment losses), it provides a relative value of losses as a function of the wavelength. Therefore, the experimental values show the real optimized wavelength (524 nm), which is 21 nm shorter than the theoretical wavelength. This can be explained by any waveguide fluctuation, roughness, or defects caused by laser inscription power fluctuations, scratches on the surface, motor vibrations, or material imperfection, which results in a lower effective bend radius. Note that the Fresnel and mode mismatch losses are wavelength-dependent but should not significantly affect the value of the obtained optimized wavelength.

As shown in Fig. 9b, for very tight bends, the wavelength is more critical. In the case where the application requires the tightest bend, the use of the Tauc law (see Eq. 5 and Fig. 2) seems to be a practical way to obtain an efficient and reliable wavelength (or a good material choice for a fixed wavelength of interest). For GeS_4_ glass, a bandgap of 464 nm (2.67 eV) is obtained. At this wavelength, the losses (1.13 dB/100 μm) are mostly due to material absorption down to a bend radius of 430 μm. For a bend radius of 375 μm, an effective loss of 1.2 dB/100 μm is calculated.

**Fig. 10:**
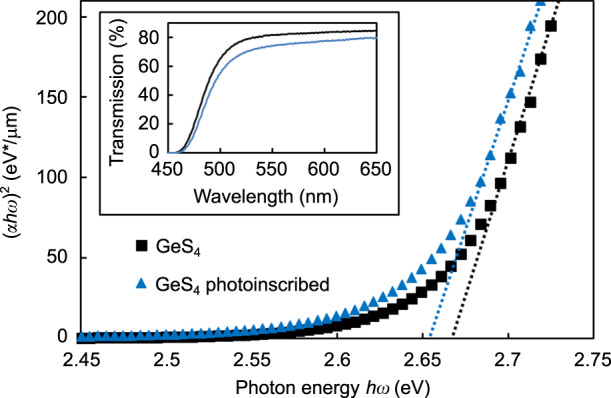
Calculating the FLIGBS value using the Tauc plot. Relationship between (*αhω*)^2^ and *hω* for a GeS_4_ glass sample before (black squares) and after (blue triangles) photoinscription. Their transmission spectra through the sample with a thickness of *d* = 1.22 mm (including Fresnel losses) are shown in the inset

As shown in Fig. 10, to obtain a lower bound of the FLIBGS in GeS_4_ glass, the same procedure using the Tauc law was executed (see section “FLIBGS theory and experiment”). The sample was irradiated from a depth of 60–660 μm over the sample with a thickness of *d* = 1.22 mm. The bandgap shifts from approximately 2.67–2.655 eV, which corresponds to an electronic resonance shift of *dλ*_1_ = 2.62 nm. The lower refractive index of GeS_4_ (2.1089 at 633 nm) makes deeper writing feasible, which probably contributes to the larger calculated band-gap shift compared with the shift for ZnSe. Unfortunately, since no Sellmeier coefficients were found in the literature for GeS_4_ glass, the theoretical curve of the refractive index contrast as a function of the wavelength could not be plotted in Fig. 6. As a comparison, a band-gap shift of approximately 0.06 eV (*dλ*_1_ ∼10 nm) was observed after illuminating a GeS_2.33_ film for 4 h using a 400-W high-pressure Hg lamp^48^. One may notice a surprising increase in the absorption in the full spectrum for the irradiated samples compared with that of the pristine samples (see the inset in Figs. 2 and 10). This is due to the light scattered from the non-uniformly inscribed sample, which is not detected by the Cary detector. To ensure that this scattered light did not affect the band-gap shift calculation, a few measurements were performed using a detector close to the sample to measure all of the scattered light, which provided the same results but with a higher experimental error. These measurements also ensured that the laser inscription did not induce significant absorption loss, which was also demonstrated by Tong et al.^49^.

## Discussion

The origin of the FLIBGS is complex and depends on the irradiated material. In glasses, the network consists of a disordered arrangement of structural units such as tetrahedra (e.g., [SiO_4_] or [GeS_4_] in silica or germanium sulfide glasses, respectively), with the existence of free space and local defects. This network therefore provides favorable conditions for material modifications under an external stimulus such as fs-laser pulses. On the other hand, in a crystalline material (e.g., ZnSe), the structure is well organized without free space and has much fewer defects than glasses. This structure then has fewer degrees of freedom for photoinduced modifications. Nevertheless, if the amplitude of photosensitivity that distinguishes these two materials is not considered, the nature of the photoinduced changes is similar. Most of the time, the photoinduced changes are a combination of two or more of the following effects: the formation of color centers, the migration of species, the modification of structural units (bond or bonding angle that breaks or changes), and even crystallization or amorphization^1,6,7^. These phenomena then result in a highly localized contraction/dilatation of the structure (i.e., a local density increase or decrease) locally altering the electron density and thus the energy required to cross the bandgap. Although the origin of these phenomena remains complex, the phenomena are generally associated with a band-gap shift (also called a transmission or absorption edge shift, photodarkening or photobleaching, or an electronic resonance shift). This is also in agreement with previously reported band-gap increases due to a decrease in lattice spacing in a semiconductor under hydrostatic pressure^50^ and using the piezospectroscopic effect^37^. Moreover, an absorption edge shift has been observed in chalcogenide glasses after illumination whose energy equals or exceeds the band-gap energy^51^. Light-induced creation of dangling bonds (immobilized free radicals) has been considered to be the origin of the phenomenon^52,53^. Recently, the creation of high-density dangling bonds after pulsed-laser excitation has been observed in hydrogenated amorphous silicon^54,55^, which could partly explain the FLIGBS. Several models have been proposed to describe the mechanisms involved in the creation of dangling bonds under illumination with energy exceeding the band-gap energy, but this issue is still controversial^51,56^. Moreover, from the illumination of chalcogenide glasses under near-band-gap light (e.g., a Hg lamp), the evidence suggests that the observed band-gap shift is due to an increase in structural intermediate-range disorder (randomness)^48,57,58^. This structural randomness may broaden the resonance frequency band. Similarly, the naturally random amorphous state of a material generally has a lower band-gap energy than its crystalline state^59,60^. This latter explanation may have an important impact on the band-gap shift in crystals, in which the structure becomes locally disordered under fs-laser illumination. Finally, despite these explanation attempts, the origins of the band-gap shift are still unclear^61^.

One can note the unusual behavior of the refractive index contrast for different laser pulse energies at the same wavelength in Figs. 3 and 6. In Fig. 6, at low energy, it is observed that the refractive index contrast increases with increasing pulse energy, whereas at higher energy, the refractive index contrast decreases. This behavior was reported in a previous work^46^ and explained by a saturation point of the refractive index change that occurs when the size of the waveguides surpasses the dimension of the fs-laser-induced plasma during the inscription. In the experiments presented in Fig. 6, the waveguide sizes surpassing the plasma size are denoted by squares (circle otherwise). The behavior follows the previous observation^46^, and the maximum refractive index contrast occurs for the highest energy pulse without the waveguide exceeding the plasma size, i.e., at 90 nJ. As shown in Fig. 3, the same trend is observed in the ZnSe crystal, where the maximum refractive index contrast is obtained at 130 nJ. The refractive index change is most negative in the red part of the spectrum and most positive in the blue part. Similarly, at 170 nJ, the refractive index change is less negative in the red part of the spectrum and less positive in the blue part. However, there is no such clear trend near the inversion of the sign of the refractive index change. This is probably due to the nonlinear nature of the refractive index change mechanisms, which is supported by the disordered refractive index profile shown in Fig. 5.

Finally, we have demonstrated an exponential increase in the photoinduced refractive index contrast for propagating wavelengths approaching electronic resonances. Unveiled by the Kramers–Kronig relations, this increase is caused by a FLIBGS in the irradiated region of transparent materials. For each material and laser, several writing parameters must be tuned to form a strong waveguide (far from resonance). In this paper, strong waveguides were not the scope of the work, and only the pulse energy was tuned to obtain decent waveguides to study the effects of an FLIBGS. Therefore, it would be of great interest to study the effects of an FLIBGS on known strong recipes and observe how the FLIBGS can push the limits of refractive index contrast and the waveguide bend radius. Exploring FLIBGS applications opens up great research opportunities for the entire spectral range in photonics, since electronic band gaps lying in the ultraviolet, visible, and infrared regions can be found in different materials.

## Materials and methods

### Refractive index modification measurement

To measure the photoinduced refractive index modifications, the structures were examined using a bright-field microscope (Olympus IX71) and a camera equipped with a bidimensional Hartmann grating (Phasics SID4Bio). The camera system acts as a wavefront analyzer that uses lateral shearing interferometry (QWLSI) to generate a quantitative phase image of transparent objects^62^. This methodology, described in detail in ref. ^63^, was carried out to recover the refractive index change (Δ*n*) of the waveguides from the phase image. Accordingly, Δ*n* measurements were considered to be exact within a 2% error margin or better. Since the Phasics camera operates in the visible range, ZnSe crystal and GeS_4_ glass, both of which have electronic bandgaps in the visible range, are excellent materials for the experiment.

### Samples

Germanium sulfide (GeS_4_) glass samples were fabricated in-house following conventional melting–quenching techniques^46^. The polycrystalline ZnSe sample was obtained from a commercial supplier (Mellers Optic).

## Supplementary information

Supplementary vedio

## References

[1] Gattass RR, Mazur E (2008). Femtosecond laser micromachining in transparent materials. Nat. Photonics.

[2] Malinauskas M (2016). Ultrafast laser processing of materials: from science to industry. Light.

[3] Eaton SM (2011). High refractive index contrast in fused silica waveguides by tightly focused, high-repetition rate femtosecond laser. J. Noncryst. Solids.

[4] Arriola A (2013). Low bend loss waveguides enable compact, efficient 3D photonic chips. Opt. Express.

[5] Chen F, De Aldana JRV (2014). Optical waveguides in crystalline dielectric materials produced by femtosecond-laser micromachining. Laser Photonics Rev..

[6] Tan DZ (2016). Femtosecond laser induced phenomena in transparent solid materials: fundamentals and applications. Prog. Mater. Sci..

[7] Fernandez TT (2018). Bespoke photonic devices using ultrafast laser driven ion migration in glasses. Prog. Mater. Sci..

[8] Gross S (2012). Ultrafast laser inscription in soft glasses: a comparative study of athermal and thermal processing regimes for guided wave optics. Int. J. Appl. Glass Sci..

[9] Lapointe J (2016). Fabrication of ultrafast laser written low-loss waveguides in flexible As_2_S_3_ chalcogenide glass tape. Opt. Lett..

[10] Okhrimchuk AG (2005). Depressed cladding, buried waveguide laser formed in a YAG: Nd^3+^ crystal by femtosecond laser writing. Opt. Lett..

[11] Liao Y (2008). Electro-optic integration of embedded electrodes and waveguides in LiNbO_3_ using a femtosecond laser. Opt. Lett..

[12] Burghoff J (2006). Efficient frequency doubling in femtosecond laser-written waveguides in lithium niobate. Appl. Phys. Lett..

[13] Poumellec B (2011). Modification thresholds in femtosecond laser processing of pure silica: review of dependencies on laser parameters [Invited]. Optical Mater. Express.

[14] Zhang BL (2011). Macroscopic invisibility cloak for visible light. Phys. Rev. Lett..

[15] Cortés LR (2018). Full-field broadband invisibility through reversible wave frequency-spectrum control. Optica.

[16] Pendry JB, Schurig D, Smith DR (2006). Controlling electromagnetic fields. Science.

[17] Leonhardt U (2006). Optical conformal mapping. Science.

[18] Charles N (2012). Design of optically path-length-matched, three-dimensional photonic circuits comprising uniquely routed waveguides. Appl. Opt..

[19] Levinshtein, M., Rumyantsev, S. & Shur, M. S. *Handbook Series on Semiconductor Parameters* (Singapore New Jersey: World Scientific, 1996).

[20] Beresna M, Gecevičius M, Kazansky PG (2014). Ultrafast laser direct writing and nanostructuring in transparent materials. Adv. Opt. Photonics.

[21] Sundaram SK, Mazur E (2002). Inducing and probing non-thermal transitions in semiconductors using femtosecond laser pulses. Nat. Mater..

[22] Chan JW (2001). Structural changes in fused silica after exposure to focused femtosecond laser pulses. Opt. Lett..

[23] Juodkazis S (2006). Laser-induced microexplosion confined in the bulk of a sapphire crystal: evidence of multimegabar pressures. Phys. Rev. Lett..

[24] Sakakura M (2007). Observation of pressure wave generated by focusing a femtosecond laser pulse inside a glass. Opt. Express.

[25] Lucarini, V. et al. *Kramers–Kronig Relations in Optical Materials Research* (Springer, Berlin, 2005).

[26] Davis KM (1996). Writing waveguides in glass with a femtosecond laser. Opt. Lett..

[27] Hirao K, Miura K (1998). Writing waveguides and gratings in silica and related materials by a femtosecond laser. J. Noncryst. Solids.

[28] Mao SS (2004). Dynamics of femtosecond laser interactions with dielectrics. Appl. Phys. A.

[29] Streltsov AM, Borrelli NF (2002). Study of femtosecond-laser-written waveguides in glasses. J. Optical Soc. Am. B.

[30] Lucarini, V. et al. *Kramers-Kronig Relations in Optical Materials Research*. (Springer, Berlin, 2005).

[31] Korff SA, Breit G (1932). Optical dispersion. Rev. Mod. Phys..

[32] Ghosh G (1997). Sellmeier coefficients and dispersion of thermo-optic coefficients for some optical glasses. Appl. Opt..

[33] Marple DTF (1964). Refractive index of ZnSe, ZnTe, and CdTe. J. Appl. Phys..

[34] Lin G (2011). Different refractive index change behavior in borosilicate glasses induced by 1 kHz and 250 kHz femtosecond lasers. Optical Mater. Express.

[35] Du X (2015). Femtosecond laser induced space-selective precipitation of a deep-ultraviolet nonlinear BaAlBO_3_F_2_ crystal in glass. J. Noncryst. Solids.

[36] Tauc J (1968). Optical properties and electronic structure of amorphous Ge and Si. Mater. Res. Bull..

[37] Akimov AV (2006). Ultrafast band-gap shift induced by a strain pulse in semiconductor heterostructures. Phys. Rev. Lett..

[38] Macdonald JR (2010). Ultrafast laser inscription of near-infrared waveguides in polycrystalline ZnSe. Opt. Lett..

[39] Tatian B (1984). Fitting refractive-index data with the Sellmeier dispersion formula. Appl. Opt..

[40] Lapointe J (2014). Making smart phones smarter with photonics. Opt. Express.

[41] Lapointe J (2015). Toward the integration of optical sensors in smartphone screens using femtosecond laser writing. Opt. Lett..

[42] Eaton SM (2005). Heat accumulation effects in femtosecond laser-written waveguides with variable repetition rate. Opt. Lett..

[43] Jesacher A (2010). Adaptive optics for direct laser writing with plasma emission aberration sensing. Opt. Express.

[44] Lapointe J, Kashyap R (2017). A simple technique to overcome self-focusing, filamentation, supercontinuum generation, aberrations, depth dependence and waveguide interface roughness using fs laser processing. Sci. Rep..

[45] Bérubé JP (2019). Femtosecond laser inscription of depressed cladding single-mode mid-infrared waveguides in sapphire. Opt. Lett..

[46] Bérubé JP (2014). Tailoring the refractive index of Ge-S based glass for 3D embedded waveguides operating in the mid-IR region. Opt. Express.

[47] Snyder, A. W. & Love, J. *Optical Waveguide Theory*. (Springer Science & Business Media, New York, 1983).

[48] Shimizu T (1978). Photo-induced ESR and optical absorption edge shift in amorphous Ge-S films. Solid State Commun..

[49] Tong L (2006). Optical loss measurements in femtosecond laser written waveguides in glass. Opt. Commun..

[50] Neuberger, M. *Handbook of Electronic Materials: Volume 5: Group IV Semiconducting Materials*. (Springer Science & Business Media, New York, 2012).

[51] Singh, J. & Shimakawa, K. *Advances in Amorphous Semiconductors*. (CRC Press, London, 2003).

[52] Hirabayashi I, Morigaki K, Nitta S (1980). New evidence for defect creation by high optical excitation in glow discharge amorphous silicon. Jpn. J. Appl. Phys..

[53] Dersch H, Stuke J, Beichler J (1981). Light‐induced dangling bonds in hydrogenated amorphous silicon. Appl. Phys. Lett..

[54] Ogihara C (2002). Lifetime and intensity of photoluminescence after light induced creation of dangling bonds in a-Si: H. J. Noncryst. Solids.

[55] Morigaki K (2003). Light-induced defect creation under pulsed subbandgap illumination in hydrogenated amorphous silicon. Philos. Mag. Lett..

[56] Morigaki, K. *Physics of Amorphous Semiconductors*. (World Scientific Press, London, 1999).

[57] Pfeiffer G, Paesler MA, Agarwal SC (1991). Reversible photodarkening of amorphous arsenic chalcogens. J. Noncryst. Solids.

[58] Street RA (1977). Non-radiative recombination in chalcogenide glasses. Solid State Commun..

[59] Feltz, A. *Amorphous Inorganic Materials and Glasses.* (VCH, Weinheim, 1993).

[60] Stuke J (1970). Review of optical and electrical properties of amorphous semiconductors. J. Noncryst. Solids.

[61] Kasap, S. & Capper, P. *Springer Handbook of Electronic and Photonic Materials*. (Springer, Cham, 2017).

[62] Roberts A (2002). Refractive-index profiling of optical fibers with axial symmetry by use of quantitative phase microscopy. Opt. Lett..

[63] Bélanger E (2018). Comparative study of quantitative phase imaging techniques for refractometry of optical waveguides. Opt. Express.

